# Effects of evidence-based nursing combined with enhanced recovery after surgery on shoulder joint function and neurological function after arthroscopic rotator cuff injury repair

**DOI:** 10.1097/MD.0000000000027951

**Published:** 2021-11-24

**Authors:** Lu He, Yanlin Li, Xinyu Liao, Yang Wang, Li Pu, Fei Gao, Guoliang Wang

**Affiliations:** Department of Sports Medicine, First Affiliated Hospital of Kunming Medical University, Kunming, Yunnan, China.

**Keywords:** arthroscopy, enhanced recovery after surgery, exercise therapy, postoperative complications, rotator cuff injurys

## Abstract

To explore the effect of Enhanced Recovery After Surgery (ERAS) nursing combined with limbs training on shoulder joint range of motion and neurological function of patients with rotator cuff injury after surgery.

60 patients who underwent arthroscopic rotator cuff repair were randomly divided into experimental group and control group, with 30 cases in each group. The experimental group received ERAS nursing combined with rehabilitation training, while the control group received routine nursing. The prognostic effects of nursing care and shoulder joint range of motion between the two groups were compared.

There were differences in general indicators between the two groups (*P* = .001). There was no significant difference in the evaluation indexes of the two groups of patients (*P* > .05). The visual analog scale score and the degree of swelling of the affected limb of the experimental group were lower than those of the control group (*P* = .001; .001). After 1, 6, 12 weeks of treatment, the Constant-Murley, American Shoulder and Elbow Surgeons and University of California-Los Angeles scores of the experimental group were higher than those of the control group (*P* = .001; .001; .001). After 2, 4 weeks of treatment, the National Institutes of Health Stroke Scale scores of the experimental group were lower than those of the control group (*P* = .001). The self-efficacy evaluation of the experimental group was significantly better than that of the control group (*P* = .001); the complication rate was lower than that of the control group (*P* = .006).

Compared with simple postoperative nursing recovery, ERAS nursing combined with limbs training can improve the exercise capacity of the shoulder joint and the recovery of neurological function, reduce the occurrence of complications.

## 1 Introduction

As one of the common shoulder joint diseases in clinic, rotator cuff injury is mostly caused by trauma and degeneration, with the tear size of about 5 cm. Shoulder pain, weakness and muscle atrophy are its main clinical symptoms, which can significantly reduce the daily quality of life of patients, and surgery is often required in the end.^[1,2]^ In recent years, with the research and development of surgical instruments and the development of arthroscopic technology, shoulder arthroscopic surgery has the advantages such as less trauma, definite efficacy and quick postoperative recovery in clinical practice compared with traditional open surgery methods, and has gradually become a more commonly used surgical method for the treatment and repair of rotator cuff injury in clinical work.^[3]^ However, arthroscopic rotator cuff repair (ARCR) has always been considered as one of the orthopedic surgeries that cause severe pain. Studies have shown that more than 50% of patients after shoulder arthroscopic surgery have experienced moderate and severe postoperative intense pain; however, improper postoperative pain management will result in prolonged pain, decreased patient satisfaction, and even dysfunction.^[3,4]^ Therefore, proper postoperative pain management for all patients after ARCR is conducted to reduce postoperative pain and improve postoperative shoulder joint function, which have become the key indicators to evaluate the success of the surgery.

Enhanced Recovery After Surgery (ERAS) refers to the concept that through a series of standardized and evidence-based nursing measures, such as optimal anesthesia methods, minimally invasive techniques, best postoperative analgesia measures, perioperative nutritional support, early guidance of patients to get out of bed and early rehabilitation exercises, the corresponding synergistic effect can be generated to minimize physiological and psychological trauma of surgical patients, reduce perioperative stress level of patients as well as the occurrence of complications, thus achieving the goal of rapid recovery.^[5,6]^ Through multi-mode and interdisciplinary nursing plan, the overall complications and length of stay can be reduced. However, there are few clinical studies on patients with rotator cuff injury by ERAS nursing combined with limbs and trunk training. After arthroscopic treatment, patients with rotator cuff injury often suffer from pain and lack of self-rehabilitation exercise after surgery, resulting in limited shoulder movement and shoulder joint adhesion in the later period, which seriously affects the efficacy. In this study, ERAS nursing combined with limbs and trunk training concept was applied to patients with ARCR, and its postoperative joint function recovery and clinical effect were discussed and analyzed.

## 2 Materials and methods

### 2.1 Patient selection and general information

60 patients with rotator cuff injury who underwent arthroscopic single-row anchor repair technique from October 2017 to October 2019 were selected. They were randomly divided into two groups, namely experimental group and control group. The experimental group received ERAS nursing combined with limb rehabilitation training, while the control group received routine nursing.

Inclusion criteria: unilateral rotator cuff tear of shoulder joint confirmed by clinical physical examination, imaging examination and intraoperative measurement; all patients were treated with arthroscopic single-row anchor repair technique. All patients had normal cognitive ability and no mental illness; there was complete follow-up clinical data. Exclusion criteria: other types of shoulder joint diseases, such as frozen shoulder; patients with a history of epilepsy and mental illness; patients who quit the study or lost contact. All patients and their family members gave informed consent to this study and signed an informed consent form, which was approved by the hospital ethics committee ((2017) ethics L NO.09).

### 2.2 Methods

Control group: Patients received routine nursing before and after routine surgery, and positive psychological counseling and preoperative education were given before surgery; skin preparation; fasting and drinking for 8 hour before surgery; anesthesia was used during the surgery, catheter and analgesic pump were indwelled after the surgery, analgesics were given as needed, pillow was removed and lying down after the surgery; after no nausea and vomiting, the diet gradually transited from liquid diet to normal diet; after surgery, the affected shoulder joint was ice compressed, and then fixed with shoulder abduction brace, and the shoulder joint flexion and extension were routinely guided.

Experimental group: Patients received ERAS nursing and limbs and trunk training from admission to discharge after surgery. A medical team that was composed of a sports medicine doctor, a head nurse, a psychologist and several nurses was established. The medical team collected the basic information of patients and the progress of nursing rehabilitation, and established good nurse-patient communication and medical communication; the nursing knowledge of rotator cuff injury was learned together, and ERAS nursing and limbs and trunk training programs was formulated, which were implemented by professional medical teams.

Preoperative nursing measures: (1) Before operation, the psychology, mental state and basic diseases of patients were comprehensively evaluated; routine examination (chest X-ray, ECG, biochemical routine, etc.) was improved; the anteroposterior and lateral radiographs, supraspinatus outlet radiographs, axillary radiographs and MRI examination of the affected shoulder joint were improved, the shoulder joint function was evaluated, and the best surgical path was established. (2) 1 day before surgery and 2 h before surgery, psychological counseling was conducted twice according to the different psychological states of the patients to eliminate the patients’ anxiety and fear about the surgery, and informed consent for surgical treatment was signed. At the same time, the responsible nurse used the Visual analogue scale (VAS) to evaluate the pain of patients, so as to guide accurate analgesia during perioperative period, keep the patients emotionally stable and actively cooperate with the medical work. (3) Wash and shave the surgical area one day before surgery to reduce the risk of infection and to give oral Lactulose Oral Solution to promote defecation. (4) Intravenous infusion of cefazolin sodium (Shenzhen CR Jiuxin Pharmaceutical Co., Ltd., NMPN H20051245) 2 g/normal saline 100 ml (Kunming Nanjiang Pharmaceutical Co., Ltd., NMPN H53020468) 2 h before surgery; If the patient was allergic to cephalosporins, 0.6 g clindamycin (Chongqing Lummy Pharmaceutical Co., Ltd., NMPN H19991072)/100 ml normal saline could be given to prevent infection.

Nursing measures during and after surgery: (1) Rehydration, keeping warm and monitoring vital signs during surgery; at the end of the surgery, 50 ml Tranexamic Acide and Sodium Chloride Injection (Chengdu Brilliant Pharmaceutical Co., Ltd., NMPN H20030625), 5 ml Ropivacaine Hydrochloride Injection (Qilu Pharmaceutical Co., Ltd., NMPN H20052716) and 1 ml Compound Betamethasone Injection (Hangzhou MSD Pharmaceutical Co., Ltd., NMPN J20140160) could be injected into the joint cavity and around the joint to relieve postoperative pain. (2) Nutritional support: Postoperative patients have no symptoms such as nausea and vomiting, they were encouraged to eat high-energy, high-protein and high-vitamin liquid food normally. (3) Pain management: intermittent ice compress was applied to the affected wound 24 to 48 hour after surgery; in order to relieve postoperative pain and local swelling of patients, and avoid adverse conditions such as wound infection and local frostbite. After surgery, 60 mg etoricoxib (Hangzhou MSD Pharmaceutical Co., Ltd., NMPN J20180057) was given orally every day for 7 days. According to the VAS, the medical staff evaluated the postoperative pain degree of the patients, made records and implemented corresponding analgesic care for the patients according to the pain degree of the patients. (4) Prevention of infection: After the surgery, the wound shall be bandaged under pressure to keep the wound dressing clean and dry. Application of broad-spectrum antibiotics to prevent infection after surgery. (5) Protect neurological function, closely observe upper limb motor function after surgery.

Functional training of affected limbs after surgery: The experimental group added limbs and trunk training on the basis of ERAS nursing. The nursing plan of this group was guided and implemented for 3 months after surgery. Before operation, the patients were instructed to be familiar with the application of shoulder brace, exercise of shoulder joint ROM, and learn reasonable and safe elbow wrist joint movement and training methods of static contraction of biceps brachii. (1) On the first day after surgery, professional rehabilitation teachers would guide patients to start fist clenching exercises and elbow flexion exercises; Patients were trained with passive traction of shoulder joint abduction and forward flexion to 90, 20 times each time and 2 groups every day. (2) From the second day to the sixth week after the surgery, the shoulder joint muscles of the patient should not exert themselves. Others should assist in completing the passive activity exercises of the affected limb. Fist clenching, shoulder shrugging, passive elbow flexion and extension exercises shall continue. Passive abduction and forward flexion of the shoulder joint should be increased to 120 degrees. Each group should be trained 20 times and 2 to 3 groups should be trained daily. (3) 6 to 8 weeks after surgery, shoulder brace could be removed during sleep, but brace must be worn during daily activities. After 6 weeks, active assistance and passive activities were carried out on the shoulder joint, and joint ROM in all directions was gradually increased, with 20 training times each time and 2 groups trained every day. (4) After 9 to 12 weeks, the brace could be removed, and the joint ROM was basically identical with that of the healthy side. The patients could use dumbbells and elastic belts to carry out resistance strength training, from small to large, step by step, try to restore the upper limb motor function, increase the strength of rotator cuff muscle training, then expand the rotator cuff tension, and enhance proprioception and flexible coordination training. Each group exercises last for 30 times, with 2 to 3 groups every day.

Postoperative psychological counseling: Due to the sudden rotator cuff injury, patients were often accompanied by anxiety, dysphoria or depression due to pain and dysfunction. The psychological state of the two groups of patients was evaluated before surgery. (1) Make individualized nursing plans according to the age, educational level and living environment of patients before surgery; through effective communication, cognition of patients for basic condition and treatment method could be increased, and their bad mood and treatment confidence could be improved. (2) Through appropriate use of analgesic drugs after surgery, medical staff explain the purpose and method of postoperative rehabilitation training to patients and their families, so as to eliminate the doubts of patients for professional knowledge and increase the compliance of patients. (3) The successful cases were introduced after the operation. Through exchanging experience, patients could enhance their confidence in rehabilitation.

### 2.3 Evaluation of nursing efficacy

The general indexes of operation, pain degree score, shoulder joint function and nerve function were taken as the main evaluation indexes; The quality of life score and mental state evaluation were used as the main evaluation index table and the incidence of complications as the secondary evaluation index to evaluate the prognosis of the two groups of patients.

#### 2.3.1 General indexes after surgery

First urination time after surgery, postoperative bed rest time, the time of eating, hospital stay and hospitalization expense were observed in the two groups.

#### 2.3.2 Pain score

Visual analogue scale was used to evaluate and compare the pain scores of patients in the two groups before surgery, 1 day, 3 days, 7 days, and 1 month after surgery. The score is between 0 and 10, 0 indicates painless, 10 indicates severe pain, and the higher the score, the more severe the pain.

#### 2.3.3 Limb swelling score

Swelling score refers to soft tissue swelling grading standard, 0 point: limb skin has no swelling, skin elasticity is normal; 1 point: limb skin is slightly swollen compared with normal skin, dermatoglyphics still exist. Compared with healthy side; 2 points: skin tension is increased compared with normal skin, dermatoglyphics disappear, there is no tension blister; 3 points: Skin swelling of limbs was obvious, higher skin temperature, tension blisters appeared. The swelling score was observed and recorded at 9: 00 am every day on the 1st, 3^rd^, and 7th day after operation.

#### 2.3.4 Evaluation of shoulder joint activity function

The shoulder joint function of the two groups was evaluated by Constant-Murley shoulder joint score, American Shoulder and Elbow Association score system and University of California shoulder joint score system before operation, 1 week after operation, 1 month after operation and 3 months after operation. (1) Constant-Murley shoulder score was used to evaluate, including pain scale 15 points, muscle strength 25 points, joint range of motion 40 points, and daily life 20 points. The higher the score was, the better the shoulder joint function was. (2) American Shoulder and Elbow Surgeons (ASES) score: mainly includes patients’ self-evaluation of pain, shoulder joint stability, shoulder joint ROM, muscle strength and other items, with a total score of 100 points. The score is positively correlated with shoulder joint function. (3) The University of California-Los Angeles (UCLA) score table includes shoulder joint pain degree, shoulder joint function, anterior flexion range of motion and strength. The total score is 35points, 28 points or less are poor, 29 points to 33 points are good, 34 points to 35points are excellent.

#### 2.3.5 Evaluation of neurological function

The National Institutes of Health Stroke Scale (NIHSS) score was used to observe the degree of neurological impairment in the two groups before operation, 2 weeks after operation and 1 month after operation. The score scale included 11 indicators, with a score range of 0–42 points, and the score was inversely proportional to the degree of neurological impairment in the patients.

#### 2.3.6 Quality of life score and mental state rating scale

The activity of daily living score (Barthel index) and quality of life score (QOL) were used to evaluate the quality of life of patients before and 3 months after surgery. The higher the score, the stronger the activity of daily living. The psychological state rating scale was evaluated by SAS scale for anxiety score and SDS scale for depression score. The higher the score, the more serious the anxiety and depression of patients.

#### 2.3.7 Complications

The incidence of complications such as joint stiffness, joint swelling, incision infection and anchor loosening in the two groups during hospitalization. Incidence (%) = number of complications/total number of cases × 100.

### 2.4 Statistical analysis

SPSS 23.0 software was used to analyze the data statistically. The measurement data are expressed by ($\bar{\text{x}}+\text{s}$) or (M plus subtraction IQR); The measurement data between the two groups are compared by independent sample *T* test; The measurement data between the two groups are compared by one-way ANOVA or K-W rank sum test to compare the score differences at different time points; The counting data is expressed as [n (%)], and the comparison of the rates between the two groups adopts *X*^2^ test; Fisher exact test was used for the counting data that do not meet the chi-square test conditions; *P* < .05 means that the difference was significant.

## 3 Results

### 3.1 Comparison of general data of patients between the two groups

There was no difference in general conditions such as gender, age, average course of disease, surgical location, surgical duration, and surgical blood loss between the two groups (*P* > .05). (Table 1).

**Table 1:** Comparison of general information between the two groups (n, $\bar{\text{x}}\pm \text{s}$).

Group	Experimental group	Control group	*χ*2/*t*	*P* value
N	30	30		
Sex (case)				
Male	18	14	1.071	.301
Female	12	16		
Average age (yr)	40.70 ± 13.41	39.70 ± 13.12	0.287	.775
Mean disease course (mo)	5.60 ± 1.38	5.27 ± 1.17	0.969	.337
Rotator cuff injury size (cm)	5.64 ± 0.47	5.63 ± 0.44	0.135	.893
Surgical site (case)				
Left shoulder	13	16	0.601	.438
Right shoulder	17	14		
Operation time (h)	1.65 ± 0.19	1.67 ± 0.20	−0.345	.731
Bleeding volume (ml)	53.98 ± 6.57	57.73 ± 8.02	−1.946	.056

### 3.2 Comparison of postoperative general conditions between the two groups

Postoperative first urination time, bed rest time, eating time, hospital stay, and hospitalization expenses of the two groups were statistically significant (*P* = .001;.001;.001;.001;.001). (Table 2).

**Table 2:** Comparison of general postoperative indexes between the two groups (n, $\bar{\text{x}}\pm \text{s}$).

Group	Experimental group	Control group	T value	*P* value
N	30	30		
Postoperative first urination time (h)	6.90 ± 0.91	10.57 ± 1.23	−12.87	.000
Postoperative bed rest time (h)	7.21 ± 0.93	8.73 ± 0.88	−6.374	.000
Eating time (h)	5.52 ± 0.67	8.49 ± 0.76	−15.73	.000
Hospital stay (d)	7.73 ± 0.90	11.65 ± 1.28	−13.43	.000
Hospitalization expenses (ten thousand yuan)	1.57 ± 0.19	2.17 ± 0.26	−9.853	.000

### 3.3 Comparison of pain scores at different time points between the two groups

**Figure 1:**
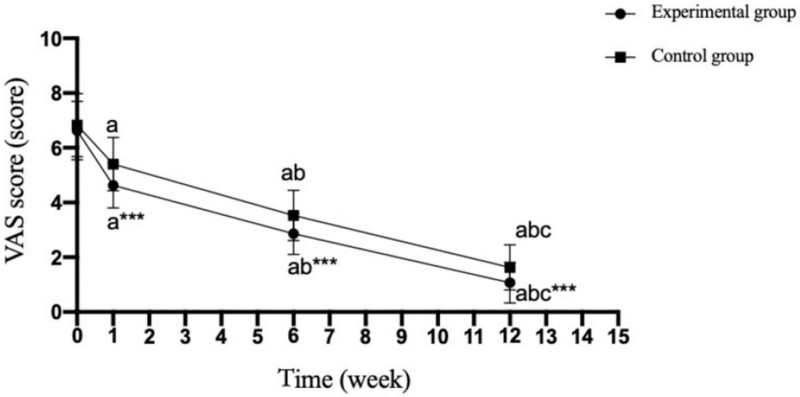
Comparison of VAS scores between the two groups at different time points. Compared with the experimental group, ^∗∗∗^*P* < .001; compared with preoperatively, ^a^*P* < .05; compared with 1 week after operation, ^b^*P* < .05; compared with 6 weeks after operation, ^c^*P* < .05. 0: before the operation; 1–15: specific weeks after the operation.

There was no significant difference in VAS score between the experimental group and the control group before surgery (*P* = .499); The VAS scores of the experimental group at 1 week, 6 weeks, and 12 weeks after surgery were significantly lower than those of the control group (*P* = .001). (Table 3 and Fig. 1).

**Table 3:** Comparison of pain scores between the two groups at different time points ($\bar{\text{x}}\pm \text{s}$, score).

Time	Experimental group	Control group	T value	*P* value
N	30	30		
Postoperative	6.63 ± 1.07	6.83 ± 1.15	−0.681	.499
1 wk after operation	4.63 ± 0.83^a^	5.40 ± 0.98^a^	−3.193	.002
6 wk after operation	2.86 ± 0.76^a^^,^^b^	3.53 ± 0.92^a^^,^^b^	−3.001	.004
12 week after operation	1.07 ± 0.740^a^^,^^b^^,^^c^	1.63 ± 0.828^a^^,^^b^^,^^c^	−2.616	.011
Experimental group F for different timepoints for	221.749			
Experimental group P for different timepoints	.000			
Control group F for different timepoints	14.308			
Control group P for different timepoints	.000			

Compared with before the operation.

a *P* < .05; Compared with 1 week after the operation.

b *P* < .05; Compared with 6 week after the operation.

c *P* < .05.

### 3.4 Comparison of postoperative swelling degree of affected limbs between the two groups at different time points

There was no significant difference between the swelling degree of the experimental group and the control group before surgery (*P* = .980); The swelling scores of the patients with rotator cuff injury in the experimental group were lower than those in the control group at 9: 00 am on the first day, the third day and the seventh day after surgery, and the comparison between the two groups was statistically significant (*P* = .001). (Table 4).

**Table 4:** Comparison of the swelling degree of the affected limb between the two groups at different time points (M ± IQR).

Time	Experimental group	Control group	*Z* value	*P* value
N	30	30		
Postoperative	1.00 (0.00,1.00)	1.00 (0.00,1.00)	0.001	.98
1d after operation	1.50^a^ (1.00,2.00)	2.00^a^ (1.00,3.00)	−2.192	.028
3d after operation	1.00^a^^,^^b^ (0.00,1.00)	1.50^a^^,^^b^ (0.75,2.00)	−2.816	.005
7d after operation	0.00^a^^,^^b^^,^^c^ (0.00,0.00)	0.00^a^^,^^b^^,^^c^ (0.00,1.00)	−2.229	.026
Experimental group Kruskal-Wallis H (different timepoints)	40.224			
Experimental group P for different timepoints	.000			
Control group Kruskal-Wallis H for different timepoints	40.161			
Control group P for different timepoints	.000			

Compared with before the operation.

a *P* < .05; Compared with 1d after the operation.

b *P* < .05; Compared with 3d after the operation.

c *P* < .05.

### 3.5 Comparison of shoulder joint function between the two groups at different time points

**Figure 2:**
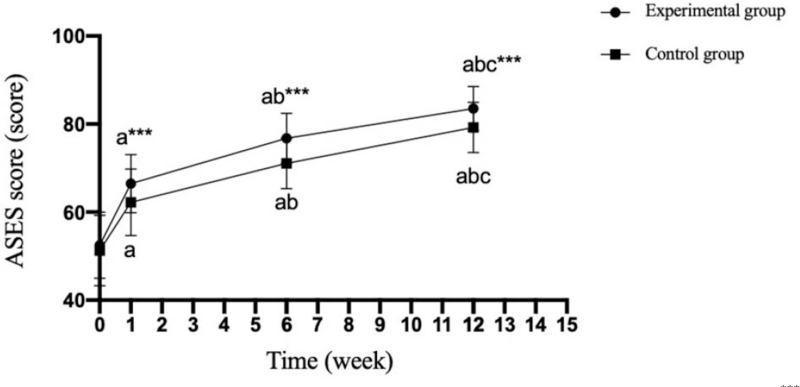
Comparison of ASES scales between the two groups at different time points. Compared with the experimental group, ^∗∗∗^*P* < .001; compared with preoperatively, ^a^*P* < .05; compared with 1 week after operation, ^b^*P* < .05; compared with 6 weeks after operation, ^c^*P* < .05. 0: before the operation; 1–15: specific weeks after the operation.

**Figure 3:**
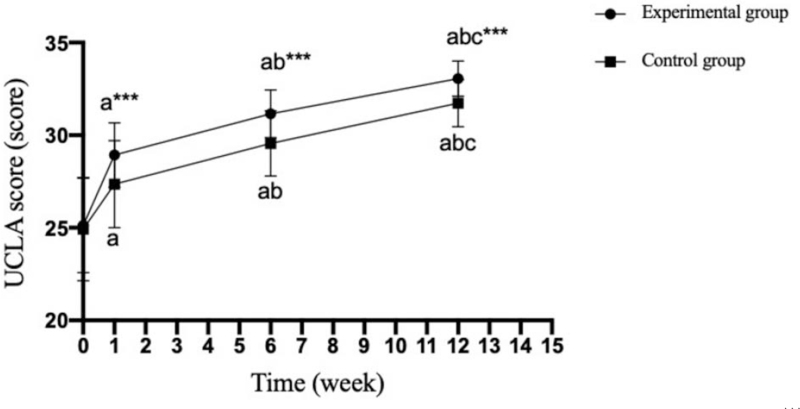
Comparison of UCLA scales between the two groups at different time points. Compared with the experimental group, ^∗∗∗^*P* < .001; compared with preoperatively, ^a^*P* < .05; compared with 1 week after operation, ^b^*P* < .05; compared with 6 weeks after operation, ^c^*P* < .05. 0: before the operation; 1–15: specific weeks after the operation.

**Figure 4:**
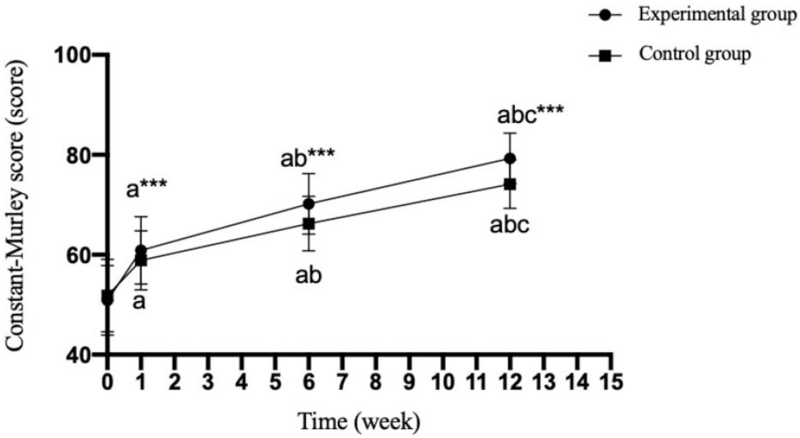
Comparison of Constant-Murley scales between the two groups at different time points. Compared with the experimental group, ^∗∗∗^*P* < .001; compared with preoperatively, ^a^*P* < .05; compared with 1 week after operation, ^b^*P* < .05; compared with 6 weeks after operation, ^c^*P* < .05. 0: before the operation; 1–15: specific weeks after the operation.

There was no significant difference in Constant-Murley, ASES and UCLA scores between the experimental group and the control group before operation (*P* = .607;.571;.777); The scores of Constant-Murley, ASES and UCLA in the experimental group were significantly higher than those in the control group at 1 week, 6 weeks and 12 weeks after operation (*P* = .001;.001;.001). (Tables 5–7 and Figs. 2–4).

**Table 5:** Comparison of the ASES scales between the two groups at different time points ($\bar{\text{x}}+\text{s}$, score).

Group	n	Postoperative	1 wk after operation	6 wk after operation	12 wk after operation
Experimental group	30	52.46 ± 7.52	66.46 ± 6.64^a^	76.76 ± 5.65^a^^,^^b^	83.50 ± 5.02^a^^,^^b^^,^^c^
Control group	30	51.3 ± 8.05	62.23 ± 7.57^a^	71.10 ± 5.75^a^^,^^b^	79.23 ± 5.72^a^^,^^b^^,^^c^
T value		0.57	2.262	3.781	3.016
*P* value		0.571	.027	.000	.004
Experimental group F for different timepoints		134.005			
Experimental group P for different time points		.000			
Control group F for different timepoints		88.670			
Control group P for different timepoints		.000			

ASES = American Shoulder and Elbow Surgeons. Compared with before the operation.

a *P* < .05; Compared with 1 week after the operation.

b *P* < .05; Compared with 6 week after the operation.

c *P* < .05.

**Table 6:** Comparison of the UCLA scale between the two groups at different time points ($\bar{\text{x}}+\text{s}$, score).

Group	n	Postoperative	1 wk after operation	6 wk after operation	12 wk after operation
Experimental group	30	25.13 ± 2.55	28.93 ± 1.74^a^	31.16 ± 1.29^a^^,^^b^	33.06 ± 0.96^a^^,^^b^^,^^c^
Control group	30	24.93 ± 2.79	27.36 ± 2.35^a^	29.56 ± 1.76^a^^,^^b^	31.73 ± 1.28^a^^,^^b^^,^^c^
T value		0.285	2.872	3.939	4.461
*P* value		.777	.006	.000	.000
Experimental group F for different timepoints		110.694			
Experimental group P for different time points		<.001			
Control group F for different timepoints		54.492			
Control group P for different timepoints		<.001			

UCLA = University of California-Los Angeles. Compared with before the operation.

a *P *< .05; Compared with 1 week after the operation.

b *P *< .05; Compared with 6 week after the operation.

c *P *< .05.

**Table 7:** Comparison of Constant-Murley scores between the two groups at different time points ($\bar{\text{x}}+\text{s}$, score).

Group	n	Postoperative	1 wk after operation	6 wk after operation	12 wk after operation
Experimental group	30	50.86 ± 6.97	60.9 ± 6.79^a^	70.16 ± 6.06^a^^,^^b^	79.26 ± 5.04^a^^,^^b^^,^^c^
Control group	30	51.83 ± 7.24	58.90 ± 5.94^a^	66.23 ± 5.47^a^^,^^b^	74.13 ± 4.86^a^^,^^b^^,^^c^
T value		−0.517	2.089	2.592	3.945
*P* value		.607	.041	.012	.000
Experimental group F for different timepoints		111.664			
Experimental group P for different timepoints		<.001			
Control group F for different timepoints		76.630			
Control group P for different timepoints		<.001			

Compared with before the operation.

a *P *< .05; Compared with 1 week after the operation.

b *P *< .05; Compared with 6 week after the operation.

c *P *< .05.

### 3.6 Comparison of shoulder joint neurological function between the two groups at different time points

**Figure 5:**
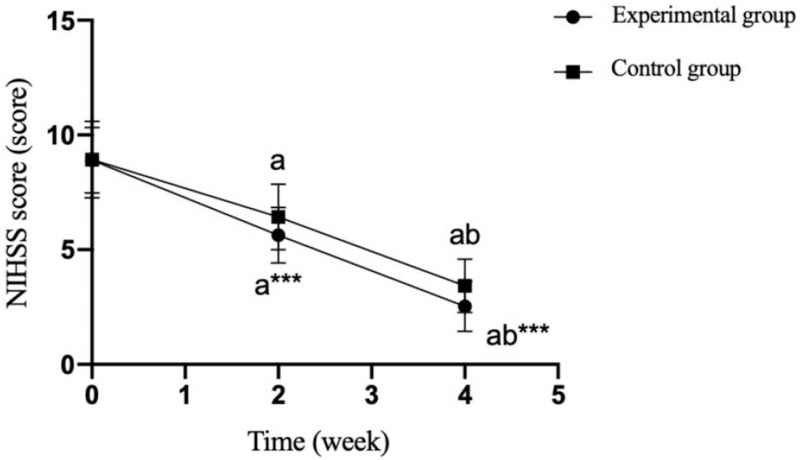
Comparison of NIHSS scores between the two groups at different time points. Compared with the experimental group, ^∗∗∗^*P* < .001; compared with preoperatively, ^a^*P* < .05; compared with 2 weeks postoperatively, ^b^*P* < .05. 0: before the operation; 1–5: specific weeks after the operation.

There was no significant difference in NIHSS scores between the two groups before surgery (*P* = .935). After 2 weeks and 4 weeks of treatment, the NIHSS scores of the two groups decreased compared with those before treatment, and the NIHSS scores of the experimental group were significantly lower than those of the control group, and the difference was statistically significant (*P* = .001). (Table 8 and Fig. 5).

**Table 8:** Comparison of NIHSS scores between the two groups at different time points ($\bar{\text{x}}+\text{s}$, score).

Group	n	Postoperative	2 wk after operation	4 wk after operation
Experimental group	30	8.9 ± 1.42	5.63 ± 1.22^a^	2.53 ± 1.08^a^^,^^b^
Control group	30	8.93 ± 1.67	6.43 ± 1.43^a^	3.43 ± 1.17^a^^,^^b^
T value			−2.288	−3.139
*P* value			.026	.003
Experimental group F for different timepoints				
Experimental group P for different timepoints				
Control group F for different timepoints				
Control group P for different timepoints				

NIHSS = National Institutes of Health Stroke Scale; Compared with before the operation.

a *P *< .05; Compared with 2 week after the operation.

b *P *< .05.

### 3.7 Comparison of quality of life and psychological state evaluation between the two groups

There were no significant differences in QOL scores, Barthel index, SAS scale and SDS scale between the two groups before surgery (*P* = .702;.954;.128;.771). After intervention, the SAS score and SDS score of the experimental group were lower than those of the control group (*P* = .001; .001); The Barthel index and QOL scores of patients in the experimental group were higher than those in the control group (*P* = .001; .001). (Table 9).

**Table 9:** Comparison of self-efficacy between the two groups.

Group	Experimental group	Control group	T value	*P* value
N	30	30		
Barthel index (score)				
Before nuring	51.36 ± 7.04	51.26 ± 5.91	0.059	.954
After nuring	78.83 ± 3.66	73.43 ± 4.99	4.694	.000
QOL score (score)				
Before nuring	51.13 ± 6.23	53.30 ± 5.98	0.385	.702
After nuring	79.86 ± 3.66	71.86 ± 4.39	7.668	.000
SAS score (score)				
Before nuring	60.86 ± 6.09	63.16 ± 5.20	−1.544	.128
After nuring	29.43 ± 3.32	33.03 ± 3.22	−4.183	.000
SDS score (score)				
Before nuring	64.03 ± 5.51	63.60 ± 5.74	0.293	.771
After nuring	29.66 ± 3.11	33.73 ± 2.90	−5.14	.000

QOL = quality of life score, SAS = self-rating anxiety scale, SDS = self-rating depression scale.

### 3.8 Comparison of the incidence of postoperative complications between the two groups

In the experimental group, there was slight joint swelling in 1 case, joint stiffness in 1 case, no incision infection or anchor loosening occurred, and it improved after symptomatic treatment, and the incidence of complications was 6.66%; In the control group, there were 3 cases with joint stiffness, 2 cases with joint swelling and 1 case with incision infection after operation, and the complication rate was 20%. The incidence of complications in the experimental group was significantly lower than that in the control group, and there was a significant statistical difference between the two groups (*P* = .006). (Table 10).

**Table 10:** Comparison of the incidence of postoperative complications between the two groups.

Group	Experimental group (n = 30) n (%)	Control group (n = 30) n (%)	χX^2^	*P* value
Joint stiffness	1 (3.33%)	3 (10%)	–	.612
Joint swelling	1 (3.33%)	2 (6.67%)	–	.98
Incision infection	0 (0.00)	1 (3.33%)	–	1.000
Anchor loosen	–	–	–	–
Total incidence	6.66%(2)	20%(6)	7.500	.006

## 4 Discussion

For the treatment of rotator cuff injury, from incision and reconstruction to arthroscopic-assisted minimally invasive repair and reconstruction, full arthroscopic minimally invasive repair and reconstruction is one of the best treatment methods at present, and its main advantages include less blood loss during surgery, less postoperative pain, less adverse reactions, convenient rehabilitation exercise and others; However, problems such as pain and joint stiffness after arthroscopic rotator cuff repair still seriously affect shoulder joint rehabilitation training.^[7]^ Therefore, the change of surgical methods and the strong demand of patients for rehabilitation training make nurses constantly improve their nursing methods and bring new challenges to nursing. ERAS nursing refers to the application of a series of evidence-based optimized nursing measures for operational intervention in all stages of perioperative period, so as to achieve the purpose of reducing surgical trauma, relieving preoperative psychological anxiety of patients, reducing surgical physiological stress reaction and postoperative complications, and improving rehabilitation effect.^[8,9]^ Rotator cuff injury not only damages the function and structure of the shoulder joint of the patient before surgery, but also affects the recovery of the shoulder joint function due to postoperative pain, inflammatory reaction caused by repair injury, tissue edema, scar formed during repair, etc., eventually leading to a decrease in patient satisfaction and an increase in length of stay, with poor functional recovery.^[10]^ ERAS nursing refers to the process of nursing staff planning nursing activities, In the process of carefully, clearly and wisely combining scientific research conclusions with clinical experience and patients’ wishes to obtain evidence as the basis for clinical nursing decision-making, the team provided professional and scientific nursing programs for patients through systematic training of nurses on rotator cuff injury disease knowledge and postoperative nursing methods. Some studies have found that ERAS nursing and limbs and trunk training can promote blood circulation of affected limbs after shoulder joint replacement, promote vigorous soft tissue metabolic function, improve tissue nutrition, enhance muscle strength, improve elasticity of ligament joint capsule, restore joint function as soon as possible, accelerate recovery speed in recovery period, reduce length of stay, reduce infection risk and improve patient satisfaction.^[11]^ In this study, there are significant differences between the two groups in the time of getting out of bed for the first time, the time of defecation for the first time after surgery, the time of hospitalization and the cost of hospitalization, and the experimental group is obviously better than the control group.

Shoulder joint is the largest and most flexible joint of upper limb, surrounded by abundant blood vessels and nerve tissue. Significant pain usually occurs after arthroscopic rotator cuff repair, but the factors leading to rotator cuff pain after surgery are still unclear. Davidson et al^[12]^ found that rotator cuff injury causes elastic retraction of fibrous tissue, thus generating greater tension. The higher the tension after repair, the more serious the postoperative pain. Therefore, it is speculated that the cause of early pain after arthroscopic rotator cuff repair is related to the great tension after repair. Some studies have shown that there are a large number of inflammatory cells and neovascularization at the edge of rotator cuff injured tendon. Neovascularization is usually accompanied by the growth of new nerve fibers in tendon, which are often important factors causing pain.^[13–15]^ Therefore, postoperative pain control is still a problem faced by surgeons and patients after surgery. Appropriate analgesia can greatly improve the patient's experience, improve the ROM of patient, and speed up the process of treatment and rehabilitation. Uquillas et al^[16]^ reviewed and compared cryotherapy, intralesional anesthesia, nerve block and other analgesic modes, and comprehensively considered the overall factors of patients, so as to provide the safest and most effective analgesic method, thus reducing adverse reactions of oral opioids, reducing complications and improving patient satisfaction. ERAS nursing can relieve patients’ anxiety and fear through preoperative health education, enhance their confidence in surgical rehabilitation, and relieve knee joint pain through postoperative analgesia, thus reducing the sensitivity of nervous system. In this study, the experimental group adopted a multi-mode analgesic scheme, giving psychological intervention before the surgery, intraoperative local anesthetic infiltration anesthesia around the joints, oral analgesics after surgery, intermittent ice compress of the affected limb to relieve postoperative pain and swelling. The comparison of postoperative VAS score and swelling scale showed that the pain score and swelling degree of the experimental group were significantly lower than those of the control group.

ARCR patients should actively implement scientific and reasonable rehabilitation training programs in the early stage. Active and passive exercises of finger joints, elbow and wrist joints can be carried out two days after surgery to restore joint movement and reduce the occurrence probability of apraxia muscular atrophy and postoperative stiffness.^[16,17]^ ROM score and ASES score are the main indexes to evaluate shoulder joint function after ARCR; The higher the ROM score and ASES score, the better the shoulder joint function. Hagen et al^[5]^ found that joint ROM such as flexion, extension and abduction in the early rehabilitation group after shoulder joint replacement was significantly improved compared with that in the delayed rehabilitation group, avoiding local tissue spasm and joint stiffness after surgery. The results of this study showed that the shoulder joint Constant-Murley score, UCLA score and ASES score of the experimental group at different times after surgery were significantly higher than those of the control group (P = 0.001; 0.001; 0.001), indicating that ERAS nursing combined with limbs and trunk training can enhance the recovery of shoulder joint function after surgery and improve the prognosis. The reason may be that early rehabilitation exercise after surgery can significantly relieve inflammatory reaction of local tissues and eliminate edema, relieve postoperative pain, accelerate tissue healing, reduce the incidence of postoperative complications, and prevent the occurrence of muscular atrophy around shoulder joint and tissue adhesion around shoulder joint. ERAS nursing combined with limbs and trunk training can avoid secondary injury of rotator cuff repaired by patients through scientific guidance of professional teams, and can gradually increase the strength of rotator cuff muscle and the stability of shoulder joint through continuous rehabilitation exercise.

NIHSS score is usually used to evaluate patients’ neurological impairment. It has the advantages of reliability, simplicity and effectiveness. It can be evaluated by non-neurologists. The higher the score, the more serious the degree of neurological impairment.^[18]^ There are many tendons around the shoulder joint, abundant nerve endings and poor blood circulation. Therefore, the pain receptors on the affected joint are extremely sensitive to pain mediators and are prone to severe pain after surgery.^[18,19]^ Early ERAS nursing and shoulder joint passive exercise can effectively improve neurological function, accelerate tendon blood circulation and lymph circulation, improve endorphin and 5-hydroxytryptamine levels, relieve pain and reduce sympathetic nerve tension. This study found that ERAS nursing combined with limbs and trunk training can significantly reduce NIHSS score, relieve pain and improve nerve injury.

Patients with rotator cuff injury are often accompanied by pain of different degrees and duration before surgery. Severe pain can lead to difficulty in falling asleep or even being unable to lie down, resulting in anxiety and depression, which seriously affects the quality of daily life of patients.^[20]^ In this study, through the implementation of health education and psychological intervention for patients with rotator cuff injury, the cognition of patients for disease can be improved, so that the patient can maintain a positive and optimistic psychology, significantly reduce and relieve the anxiety and depression of the patient, and be full of confidence in the recovery of the disease. From the research results, it can be seen that the SAS score and SDS score of the study group are significantly lower than those of the control group, the Barthel index and QOL score are higher than those of the control group, with significant differences. Joint stiffness and joint swelling are common complications in ARCR patients after surgery, which seriously affect the prognosis of shoulder joint function of patients.^[16,21]^ This study found that compared with conventional nursing in the control group, ERAS nursing combined with limbs and trunk training in the experimental group can significantly reduce the overall complication rate after ARCR and improve the postoperative efficacy and rehabilitation effect.

Compared with simple postoperative nursing recovery, ERAS nursing combined with limbs and trunk training can significantly improve the exercise capacity of the shoulder joint and the recovery of neurological function, enhance the understanding of patients for diseases and reduce the occurrence of complications.

## 5 Conclusion

In conclusion, under the guidance of ERAS nursing combined with limbs and trunk training program, perioperative nursing of arthroscopic rotator cuff repair can eliminate the tension and anxiety of patients, reduce bed rest time, effectively improve the recovery degree of shoulder joint function and quality of life of patients after surgery, and effectively reduce the incidence of postoperative complications of patients. However, the sample size included in this study is small and the follow-up time is short. The follow-up study should be to carry out long-term and multi-index postoperative nursing research on the accumulated large sample data, so as to lay a foundation for the wide promotion of later clinical nursing.

## Author contributions

**Conceptualization:** Lu He, Yanlin Li, Xinyu Liao, Fei Gao, Guoliang Wang.

**Data curation:** Lu He, Xinyu Liao, Yang Wang, Guoliang Wang.

**Formal analysis:** Lu He, Xinyu Liao, Yang Wang, Li Pu, Fei Gao, Guoliang Wang.

**Funding acquisition:** Yanlin Li, Guoliang Wang.

**Investigation:** Lu He, Li Pu, Guoliang Wang.

**Methodology:** Lu He, Xinyu Liao.

**Project administration:** Lu He, Yang Wang, Guoliang Wang.

**Software:** Fei Gao.

**Supervision:** Yanlin Li.

**Validation:** Yanlin Li, Li Pu.

**Visualization:** Fei Gao.

**Writing – original draft:** Lu He, Yanlin Li, Yang Wang, Li Pu, Guoliang Wang.

**Writing – review & editing:** Lu He, Yanlin Li, Guoliang Wang.
